# Retraction: Metal-free [2+2+1] cycloaddition polymerization of alkynes, nitriles, and oxygen atoms to functional polyoxazoles

**DOI:** 10.1039/d1ra90113c

**Published:** 2021-04-30

**Authors:** Lichao Dong, Tian Lan, Yin Liang, Shifeng Guo, Hao Zhang

**Affiliations:** The 306th Institute of China Aerospace Science & Industry Corp. Beijing 100074 China 610557405@qq.com

## Abstract

Retraction of ‘Metal-free [2+2+1] cycloaddition polymerization of alkynes, nitriles, and oxygen atoms to functional polyoxazoles’ by Lichao Dong *et al.*, *RSC Adv.*, 2020, **10**, 24368–24373, DOI: 10.1039/D0RA04249H.

We, the named authors, hereby wholly retract this *RSC Advances* article as there is insufficient data to support the structure of the final polymer product, and therefore, the conclusions are not fully supported. In Fig. 1 the peaks of the ^1^H-NMR spectrum are stacked together and therefore the spectrum cannot be used as proof of the polymer structure. In Fig. 2, the ^13^C-NMR spectrum, the peaks on the benzene ring cannot correspond to before and after polymerization. Therefore, further research and characterization work is needed to determine the structure of the resulting polymer.

The corresponding authors regret this oversight and apologise for any inconvenience to readers.

 

Signed: Lichao Dong, Tian Lan, Yin Liang, Shifeng Guo and Hao Zhang

Date: 18 April 2021

 

Retraction endorsed by Laura Fisher, Executive Editor, *RSC Advances*

## Supplementary Material

